# SimText: a text mining framework for interactive analysis and visualization of similarities among biomedical entities

**DOI:** 10.1093/bioinformatics/btab365

**Published:** 2021-05-25

**Authors:** Marie Macnee, Eduardo Pérez-Palma, Sarah Schumacher-Bass, Jarrod Dalton, Costin Leu, Daniel Blankenberg, Dennis Lal

**Affiliations:** Cologne Center for Genomics (CCG), Medical Faculty of the University of Cologne, University Hospital of Cologne, Cologne 50931, Germany; Universidad del Desarrollo, Centro de Genética y Genómica, Facultad de Medicina Clínica Alemana, Santiago 7590943, Chile; Lerner Research Institute, Cleveland Clinic, Cleveland, OH 44195, USA; Department of Cardiovascular and Metabolic Sciences, Cleveland Clinic, Cleveland, OH, 44195, USA; Genomic Medicine Institute, Lerner Research Institute, Cleveland Clinic, Cleveland, OH 44195, USA; Genomic Medicine Institute, Lerner Research Institute, Cleveland Clinic, Cleveland, OH 44195, USA; Cologne Center for Genomics (CCG), Medical Faculty of the University of Cologne, University Hospital of Cologne, Cologne 50931, Germany; Genomic Medicine Institute, Lerner Research Institute, Cleveland Clinic, Cleveland, OH 44195, USA; Stanley Center, Broad Institute of MIT and Harvard, Cambridge, MA 02142, USA; Epilepsy Center, Neurological Institute, Cleveland Clinic, Cleveland, OH 44195, USA

## Abstract

**Summary:**

Literature exploration in PubMed on a large number of biomedical entities (e.g. genes, diseases or experiments) can be time-consuming and challenging, especially when assessing associations between entities. Here, we describe SimText, a user-friendly toolset that provides customizable and systematic workflows for the analysis of similarities among a set of entities based on text. SimText can be used for (i) text collection from PubMed and extraction of words with different text mining approaches, and (ii) interactive analysis and visualization of data using unsupervised learning techniques in an interactive app.

**Availability and implementation:**

We developed SimText as an open-source R software and integrated it into Galaxy (https://usegalaxy.eu), an online data analysis platform with supporting self-learning training material available at https://training.galaxyproject.org. A command-line version of the toolset is available for download from GitHub (https://github.com/dlal-group/simtext) or as Docker image (https://hub.docker.com/r/dlalgroup/simtext/tags.).

**Supplementary information:**

Supplementary data are available at *Bioinformatics* online.

## 1 Introduction

Researchers rely on time-intensive manual literature surveys to compare biomedical entities (e.g. genes, authors or disorders) to one another and to learn about the research landscape overall. Various tools and packages have been developed to extract higher-level information from the literature in a systematic way. Without the need for programming, different web tools and databases provide summary statistics and annotations for results of a single search term (Garcia-Pelaez *et al.*, 2019; Wei *et al.*, 2019) or associations and relationships among biomedical entities in the literature (Ren *et al.*, 2018; Szklarczyk *et al.*, 2019). However, such web tools and databases cannot be customized, do not visualize the results or are focused on specific applications (e.g. relationships among proteins). To compute similarities between entities, many recently published methods use word and concept embeddings techniques, e.g. BioBERT, as opposed to comparing raw text words (Junge and Jensen, 2020; Lee *et al.*, 2020; Szklarczyk *et al.*, 2019). Here, we focus on a different approach that can be applied to any kind of strings. Frequent words or scientific terms are extracted from text and compared among biomedical entities of interest while assuming that more similar or related biomedical entities share more frequently co-occurring words and scientific terms in their text sources than unrelated entities. Our semi-automatic framework for literature research, SimText, allows users to collect text from PubMed for any given set of biomedical entities, extract associated vocabulary and visually inspect similarities among them and their key characteristics in an interactive app. To make large-scale literature analyses accessible to everyone, also to people who do not code, we provide the SimText toolset without the requirement of installation in the online data analysis platform Galaxy.

## 2 SimText description and workflow

**Fig. 1.**
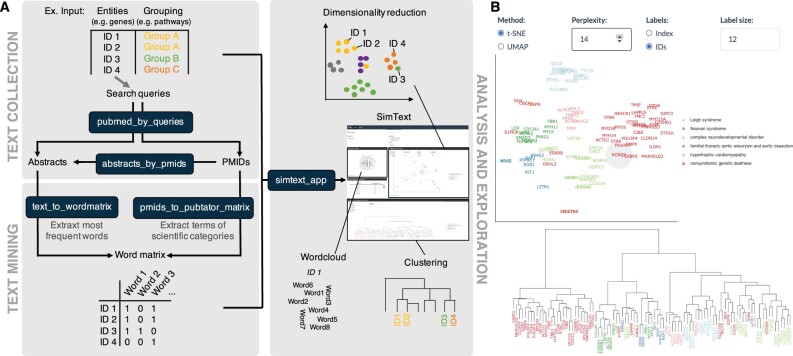
Schematic presentation of the SimText toolset. (**A**) Tools are shown in dark blue boxes. Top left: For text collection of a set of entities (e.g. gene names), the entities are provided as search queries to retrieve abstracts or PMIDs from PubMed (‘pubmed_by_queries’). Else, the user can provide manually curated PMIDs for each entity that are used to fetch the corresponding abstracts (‘abstracts_by_pmids’). Bottom left: From the collected abstracts and/or manually curated text, the corresponding vocabulary associated with each entity is extracted while providing various optional text-mining techniques (‘text_to_wordmatrix’). Alternatively, using PMIDs as input, scientific terms of specific categories can be extracted for each entity using PubTator (‘pmids_to_pubtator_matrix’). In both approaches, the output represents a binary matrix with all extracted words and entities. Right: Analysis of the generated matrix is enabled by an interactive app (‘simtext_app’). The key characteristics of the entities can be explored, and different dimension reduction and clustering techniques can be applied to the matrix to visualize similarities among the entities. Custom grouping variables (e.g. associated diseases or pathways of genes) can be compared with the grouping of the entities based on their associated vocabulary. (**B**) Dimensionality reduction plot and hierarchical clustering of monogenic disorder genes (use-case example 1) in the SimText app

SimText tools can be used individually or combined for a complete analysis, as detailed in Figure 1A. All tools are available as command-line tools or along many more text manipulation tools as part of the online data analysis platform Galaxy (Afgan *et al.*, 2018). Detailed descriptions of the tools can be found in Supplementary Material. SimText consists of the following three modules:

**Text collection:** SimText provides two tools to collect text from abstracts related to the entities of interest. Abstracts or PubMed identifiers (PMIDs) related to the entities of interest (e.g. gene names) can be retrieved automatically based on PubMed’s keyword search rules and syntax using the ’pubmed_by_queries’ tool. Using the ’abstracts_by_pmids’ tool, pre-populated PMIDs for each entity can be used to fetch the corresponding abstracts. Alternatively, custom text can be provided to be analyzed instead or in addition to the collected text.

**Text mining:** Two different vocabularies can be generated for each biomedical entity. The ’text_to_wordmatrix’ tool identifies the most frequently occurring words, after optional word quality control, from all collected text for each biomedical entity. Alternatively, the ’pmids_to_pubtator_matrix’ tool extracts scientific terms using PubTator (Wei *et al.*, 2019) annotations of biomedical concepts. For both tools, the output is a high-dimensional binary matrix of all terms and biomedical entities.

**Analysis and exploration:** In an interactive app (’simtext_app’, online in Galaxy or offline as command-line tool), groups of related biomedical entities can be analyzed and visualized by applying different unsupervised learning techniques to the matrix from the previous step (detailed in Supplementary Material).

## 3 Use-case examples

Our use-case examples are described in detail in Supplementary Material and can be reproduced using commands and data from our GitHub repository or by following our Galaxy training material. In one use-case example, we validated the approach by performing a SimText analysis on 95 monogenic disorder genes and hypothesized that the large-scale gene-level information extraction from abstracts could be used to replicate expert-curated disorder categories. In the downstream analysis in the interactive SimText app, we found that the gene grouping based on associated vocabulary is concordant with expert-curated disorder categories (Fig. 1B). To quantify this, we calculated the Adjusted Rand Index (ARI) and found a moderate (ARI = 0.6, using the ‘text_to_wordmatrix’ tool to extract frequent words) to good (ARI = 0.84, using the ‘pmids_to_pubtator_matrix’ tool to extract frequent scientific terms) agreement between the expert-curated disorder categories and the text-based gene grouping. Notably, several genes do not cluster or group with genes of their pre-existing disorder category. For example, *KCNQ4* was found in a cluster of genes associated with neurodevelopmental disorders, particularly with *KCNB1* but was pre-defined to be associated with non-syndromic genetic deafness. A literature search shows that SimText can help to identify similar genes beyond clinical phenotype grouping as both genes share similarities in biology: *KCNQ4*, as well as *KCNB1* encode potassium channels and *KCNB1* is thought to play a critical role in the regulation of neuronal excitability, particularly in sensory cells of the cochlea (Kubisch *et al.*, 1999). In another example, we systematically assessed shared (or distinct) interests among 185 researchers from 12 different departments based on the word content of their published abstracts. By visualizing the results in the SimText app we could identify groups of researchers with similar interests beyond department borders, which is valuable knowledge potentially leading to fruitful collaborations. As shown, SimText can equally extract valuable information from very different types of biological entities, such as genes and researchers. The results can be found for illustrative purposes at https://simtext.shinyapps.io/genes and https://simtext.shinyapps.io/researcher.

## 4 Conclusion

SimText enables the extraction of knowledge for large lists of biomedical entities and the visual exploration of any existing relationships among them. This way, overlooked implicit similarities and connections in the literature can be found, and hypotheses for scientific research can be generated. We demonstrate the versatility of SimText in our use-case examples, e.g. by identifying investigators with similar interests from a large multi-disciplinary research institute. The SimText tools can be used without programming knowledge nor require installation, individually or in different workflows for a large number of possible use-cases.

*Financial Support: none declared*.

##  

*Conflict of Interest*: D.B. has a significant financial interest in GalaxyWorks, a company that may have a commercial interest in the results of this research and technology. This potential conflict of interest has been reviewed and is managed by the Cleveland Clinic.

## Supplementary Material

btab365_Supplementary_Data
